# Positive Peer Groups Support Preschoolers’ Word Learning

**DOI:** 10.1111/desc.70294

**Published:** 2026-10-07

**Authors:** Elise Breitfeld, Courtney N. Snoberger, Jenny R. Saffran

**Affiliations:** ^1^ Department of Psychology University of Wisconsin‐Madison Madison Wisconsin USA

**Keywords:** language development, peer interaction, preschool, word learning

## Abstract

Children learn language from their social partners, and differences in the behavior of these social partners can influence language learning outcomes. The literature largely focuses on adult caregivers; however, there is less clarity regarding the extent to which the social behavior of peers might also impact children's language learning. Children spend a large proportion of their time with peers once they enter preschool; peers are thus an important source of input for learning. Moreover, preschoolers are not just interacting with individual peers but also with groups of peers. To explore the impact of peers’ social behavior on preschoolers’ word learning, the present study manipulated peer behavior at two levels: individual peers and the peer group context. Forty‐four preschoolers (36‐60 months) completed a tablet task in which they watched a series of animated videos of a classroom. Participants were exposed to (1) either a positive or negative social group (manipulated between‐subjects) and (2) a positive and negative individual (manipulated within‐subjects). Participants were then taught four novel object‐label pairings by the children in the video. We measured preschoolers’ accuracy in learning these novel object‐label pairs. Overall, preschoolers learned the words above chance and there was no difference in learning from the positive and negative individual peers. However, participants learned words significantly better in the positive group than in the negative group. This pattern of results suggests that peer groups, and the overall affective context of children's learning environments, may play important roles in boosting preschoolers’ language learning trajectories.

## Summary

- This study provides novel insights into how preschoolers’ social interactions with peers impact their word learning success.
- In an animated tablet task, we manipulated the social behavior of peers (positive vs. negative) at two levels: peer group (between‐subjects) and individual peers (within‐subjects).
- After manipulating peer behavior, preschoolers learned novel words; they learned better in the positive group than negative group, but equally well from the individual peers.
- The results suggest that the behavior of peer groups and preschoolers’ broader social contexts may play a key role in facilitating word learning.

## 1 Introduction

Opportunities for early language learning unfold within young children's social interactions with others. Importantly, the social dynamics of these interactions have the potential to vary widely: children interact with many different social partners within many different contexts. As such, certain social dynamics may strengthen children's likelihood of learning language while others might hinder language learning opportunities. Understanding these factors is of both theoretical and applied importance given the tremendous variance in children's social environments.

Social factors begin to influence even the earliest stages of language learning in infancy. Infants show a preference for listening to and learning from infant‐directed speech (IDS): slower, higher pitched speech with shorter utterances that is frequently used by caregivers, compared to adult‐directed speech (Eiteljoerge et al. 2025; Graf Estes and Hurley 2013; Ma et al. 2011; ManyBabies Consortium 2020). It has been suggested that this early preference for IDS is driven by the positive affect caregivers typically demonstrate when using IDS. Even when speech sounds are removed, infants prefer to look at a silent recording of a speaker using IDS compared to a speaker using ADS (Kim and Johnson 2014). Furthermore, infants’ preference for IDS over ADS disappears when IDS speakers use neutral or negative affect or when ADS speakers use positive affect (Kim and Johnson 2013; Singh et al. 2002). Thus, even in infancy, speaker affect emerges as a key factor impacting language learning.

This preference for and improved learning from speakers displaying positive affect persists into childhood. When two speakers provide conflicting labels for a novel object, preschoolers are more likely to endorse a novel label for an object provided by a speaker making a happy face compared to a conflicting label for the same object provided by a speaker making an angry face (Clément et al. 2013). Speakers’ positive affect and social behavior even influence children's preferences above and beyond other salient characteristics; while young children typically prefer native‐accented to foreign‐accented speakers, this preference flips when the native‐accented speaker is depicted as mean and the foreign‐accented speaker is depicted as nice (Kinzler and DeJesus 2013). Similarly, preschoolers weight benevolence information over expertise information about speakers when choosing whom to learn words from (Landrum et al. 2013). These expressed preferences are also reflected in children's word learning outcomes. Even when two speakers behaved identically *during* learning, toddlers learned words better from a speaker who had *previously* demonstrated infant‐directedness and positive affect compared to a speaker who had previously demonstrated adult‐directedness and neutral affect (Breitfeld et al. 2024). Therefore, not only does speaker affect during language learning opportunities matter, but speakers’ prior social behavior can also influence children's subsequent language learning outcomes.

The literature investigating young children's preferences for and learning from different sociolinguistic stimuli has focused primarily on adults as children's social partners and providers of language input. While there is no doubt that children spend a large portion of their time interacting and speaking with adult caregivers, most children also receive language input from other children. One study of the naturalistic home language environment of English‐learning toddlers found that for children with older siblings, 16% of the child‐directed language input they heard came from other children (Shneidman et al. 2013). Furthermore, including other children in measures of language input provided a better predictor of later vocabulary size compared to just considering language input from the child's primary caregiver. Once children enter preschool, about 40% of their interactions in the classroom are with other children, and children's language growth is related to the composition and language skills of peers in their class (Chaparro‐Moreno et al. 2019; Justice et al. 2011; Kohl et al. 2022; Mashburn et al. 2009). Importantly, in many cultures around the world, child‐directed input from adults is relatively infrequent, making language input from other children even more influential (Casillas et al. 2021; Cristia et al. 2019; Schick and Stoll 2025). In fact, in some cases, there is evidence that children may actually learn words better from other children than from adults (Erskine and Seidl 2026). Thus, examining the social factors, like positive and negative affect, that might impact how well preschool‐age children learn language from their peers is an important component of understanding young children's language development trajectories.

The current study is a first step towards examining the impact of peers’ social behavior on children's ability to learn words from them. Because peers become particularly important as learning partners when children enter school settings (including daycare), we took the classroom as a starting point. As we considered potential roles for peer social behavior in influencing language learning, we focused on two different levels of social interaction. The first level is the behavior of individual peers, whose affect may support or discourage word learning. The second level is the classroom itself, which may provide a more positive or more negative context for learning. We reasoned that in classroom environments, both levels (i.e., individuals and peer groups) may impact learning trajectories. Children are interacting with multiple peers, and therefore accumulate experience with the social behavior of individual peers as well as the emotional valence of the social context as a whole.

Previous work has largely only examined how the behavior of *individual* social partners impacts children's learning. However, children's social environments are made up of multiple individuals who together contribute to the broader socioemotional context. And indeed, socioemotional features of children's environments as a whole also impact cognitive processing and learning outcomes. For example, children's processing is facilitated in task contexts using positively‐valenced stimuli compared to negatively‐valenced stimuli (Ponari et al. 2018; Qu and Zelazo 2007). Relatedly, children are more likely to know words they heard within interactional contexts caregivers rated as more positively valenced (Nencheva et al. 2026). Other measures of social environment quality—like the frequency with which children experience contingent and predictable responses from their social partners—also predict learning and language development (Forest et al. 2025; Luchkina and Xu 2024). It has been suggested that these features of children's social environments support language learning by promoting attention to social partners and the language input they provide (e.g., Masek et al. 2021). We propose that social behavior from peers (both as individuals and as a group context) may similarly influence children's word learning, perhaps via related mechanisms whereby positively‐valenced stimuli boost processing and attention.

The current study manipulated two aspects of children's social experience with peers—the emotional valence of the context as well as the emotional valence of individuals’ behavior—in a word learning tablet task. To manipulate these dimensions of experience, preschoolers (3‐ to 5‐year‐olds) watched an animated video in which they were introduced to a classroom context. Half of the participants were introduced to a positive context (via the dialogue and facial expressions of multiple children in the classroom; Positive Context condition) and the other half of participants were introduced to a negative context (Negative Context condition). Next, all participants were introduced to two individual children in the class, one who behaved positively (Positive Peer) and one who behaved negatively (Negative Peer). Finally, children were taught novel words by these peers (using neutral valence) and tested on their learning. If preschoolers’ word learning is impacted by the emotional valence of the social context within which they are learning, we hypothesized that children would learn words better in the Positive Context condition than in the Negative Context condition and that, within these contexts, they would learn better from the Positive Peer than the Negative Peer. Our hypothesis and analytic approach were preregistered and can be accessed along with all stimuli, data, and analysis scripts on the Open Science Framework (https://osf.io/zswe3/overview).

## 2 Methods

### 2.1 Participants

Forty‐four 3‐ to 5‐year‐old children were recruited from an existing database of interested families or on site at the local children's museum (*M*_age_ = 48.62 months, 24 female). We chose this age group because preschool‐aged children are likely beginning to have more interactions with other children and experience in classroom contexts, and thus have at least some experience learning language from their peers. The sample size was preregistered based on an a priori power analysis in G*Power to provide 90% power to detect a medium effect size, consistent with previous investigations of preschoolers’ learning preferences (Clément et al. 2013; Kinzler and DeJesus 2013; Landrum et al. 2013). Note that G*Power provides only an approximation of the appropriate sample size for a linear‐mixed effects model approach. Eleven additional children participated but were excluded from analysis due to inattention to the task (not watching the exposure phase videos; *N* = 2), failure to complete the test trials (*N* = 6), or equipment failure (*N* = 3). All participants were primarily (75% of the time or more) English‐learners with no history of hearing or vision problems. The sample was predominantly White (89%) with 2% identifying as Asian, 2% identifying as Black or African American and 7% identifying as belonging to multiple racial categories. None of the participants identified as Hispanic. The sample was drawn from a mid‐size US city with an upper‐middle class population. Participants and their caregivers were compensated for their time and all experimental protocols were approved by the local Institutional Review Board.

### 2.2 Stimuli

The study consisted of a five‐minute animated video presented via an iPad. The video was designed specifically for this study using the GoAnimate platform (Sierksma and Shutts 2020). The word learning stimuli (see Table 1) included labels selected from the NOUN database (Horst and Hout 2016) and objects selected from the Library for Innovative Category Exemplars Database (ALICE; Xu et al. 2024).

**TABLE 1:** Selected novel words and objects.

**Label**	Fiffin	Koba	Manu	Shill
**Object**	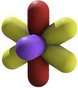	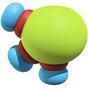	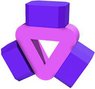	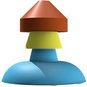

*Note*. Labels selected from NOUN database and objects selected from ALICE database.

### 2.3 Design

Participants completed a three‐phase two‐alternative forced‐choice tablet task. The task consisted of a Exposure Phase, which included both the between‐subjects Context manipulation and the within‐subjects Peer manipulation; a Learning Phase, in which participants were taught the novel object‐label pairs; and a Test Phase, in which participants’ learning of the novel object‐label pairs was tested.

#### 2.3.1 Exposure Phase

During this phase, participants watched and interacted with an animated video. In the first part of the exposure phase, a teacher introduces an art activity to a class of students. For participants in the Positive Context condition, the students in the video all respond positively. For example, students said, “*I can't wait to do art!”* and *“This is such a fun class!*” (see Figure 1a). For participants in the Negative Context condition, the students in the video all respond negatively. For example, students said, “*I hate making art.”* and *“This is such a boring class*.” The classroom portion of the video lasted one minute. Then, participants watched two individual children in the classroom have a conversation (see Figure 1b). One child provided positive facial expressions and dialogue (Positive Peer; e.g., “*Aren't you so excited to work on this art project?…*I *think we are both going to make something so cool!”*). The other child responded with negative facial expressions and dialogue (Negative Peer; e.g., “*No I am not excited, I don't like art…*I *am not good at art and I don't think you are very good at it either!*”). The full Exposure Phase dialogue can be found in the Supporting Information. The peer portion of the exposure phase also lasted one minute. Which child was the Positive Peer and which was the Negative Peer was counterbalanced across the sample. Throughout the Exposure Phase, the participant was prompted to advance the video by clicking on different items in the scene (e.g., the teacher, a table). These interactive buttons were used to provide participants with practice clicking the screen and to keep them engaged throughout the task.

**FIGURE 1:**
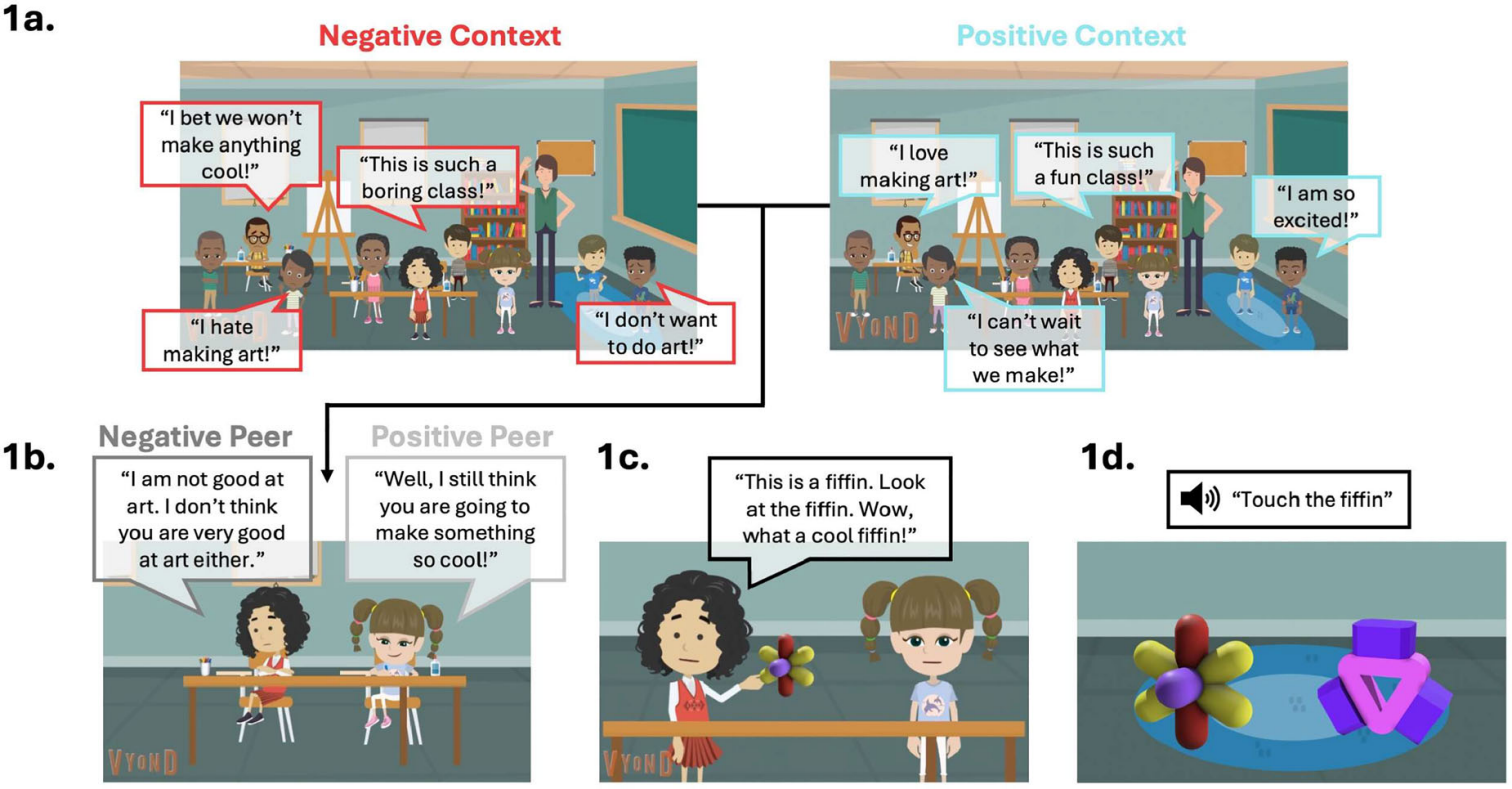
Examples of (a, b) Exposure, (c) Learning, and (d) Test phases.

#### 2.3.2 Learning Phase

During this phase, each child in the video (i.e., the Positive Peer and Negative Peer) labeled two different novel objects (four total). While the children were labeling the objects, they both displayed neutral affect (see Figure 1c). The Training Phase included eight trials: each of the four novel objects was labeled by the same child on two trials. On each trial, the object label was repeated three times (e.g., *“Look, this is a Fiffin! Do you see the Fiffin? Wow, what a cool Fiffin!”*). There were two blocks of four trials each. At the beginning of each block, each child labeled a familiar object (i.e., crayon, glue stick, paintbrush, or scissors). The assignment of novel objects to each child was counterbalanced across participants.

#### 2.3.3 Test Phase

Each participant completed 16 test trials. On each trial, participants saw a pair of novel objects from the Learning phase, one on each side of the screen (see Figure 1d). Objects were yoked such that the two objects labeled by the Positive Peer were always presented together and the two objects labeled by the Negative Peer were always together. Participants saw these pairs of objects and heard a sentence that directed them to touch one of the two objects (e.g., “*Touch the Fiffin!*”). Lastly, participants saw the Positive Peer and the Negative Peer reappear on the screen and were asked to choose which child they would prefer to play with.

### 2.4 Procedure

Participants completed the study on an iPad in a quiet room. Parents provided written informed consent, and children provided verbal assent. Caregivers were able to observe throughout the study. After the study, caregivers completed a questionnaire that included information about daycare experience and siblings. At the end of the session, the family was given time to debrief any aspects of the study.

### 2.5 Statistical Analysis

Our primary outcome measure of interest was children's accuracy on the word learning test trials. Accuracy was calculated at the participant level as a measure of the proportion of word learning trials they answered correctly. Context and Peer were effect‐coded (‐0.5 for Negative and +0.5 for Positive) and accuracy was centered around chance by subtracting 0.5 such that an accuracy value of 0 would be equal to chance performance. The primary preregistered analyses regressed word learning accuracy on Peer, on Context, and on their interaction in a linear mixed‐effects model including a by‐subject random intercept and a by‐subject random slope for Peer. However, this model with the maximal random effects structure did not converge; following recommendations for resolving convergence errors (Brauer and Curtin 2018; Muradoglu et al. 2023), the by‐subject random intercept was removed such that the random effects of the final model included only a by‐subject random slope for Peer. All analyses were run in RStudio (R version 4.3.1) using the lme4 package (version 1.1.34) and fit by maximum likelihood.

Note that a logistic regression approach (analyzing data at the trial‐level rather than the subject‐level) resolves the limitation of the reported preregistered model such that all random effects can be estimated and yields convergent results (see Supporting Information for full details). Additionally, two preregistered basic models (regressing accuracy on Context alone and regressing accuracy on Peer alone) were fit. Due to the lack of an interaction between Context and Peer, the results of the two single‐predictor models are redundant with the interaction model and therefore not reported in the main text, but can be found in the Supporting Information.

Lastly, we explored additional variables that may have influenced participants’ performance on our task by adding them as predictors to the primary linear‐mixed effects model. These variables included demographic information, like age, along with participants’ responses to the peer preference question included at the end of the test phase.

## 3 Results

To test our predictions that participants would demonstrate better word learning in the Positive Context condition than the Negative Context condition and better word learning from the Positive Peer than the Negative Peer, we regressed accuracy on Context, Peer, and their interaction in a linear mixed‐effects model including a by‐subject random slope for Peer (see Table 2).

**TABLE 2:** Linear mixed‐effects model predicting word learning accuracy.

Predictor	*b*	*SE*	*df*	*F*	*p*
Intercept	0.28	0.025	42	124.29	<0.001
Context	0.14	0.050	42	7.69	0.0082
Peer	−0.031	0.050	42	0.39	0.54
Context * Peer	0.097	0.10	42	0.93	0.34

*Note*: Model fit with a by‐subject random slope for Peer.

The intercept of this model demonstrated that children successfully learned the words above chance on average across both of our manipulations: *b* = 0.28, *SE* = 0.025, *F*(1,42) = 124.29, *p*<.001. The main effect of Context revealed that participants’ accuracy was significantly higher in the Positive Context (*M* = 0.85, *SD* = 0.17) compared to the Negative Context (*M* = 0.71, *SD* = 0.29), *b* = 0.14, *SE* = 0.050, *F*(1,42) = 7.69, *p* = 0.0082 (see Figure 2a). This pattern of results is consistent with our hypothesis that the positively‐valenced context, established by the social behavior of the peer group as a whole in the Positive Context condition, supported participants’ word learning relative to the overall negatively‐valenced social behavior of the peer group in the Negative Context condition.

**FIGURE 2:**
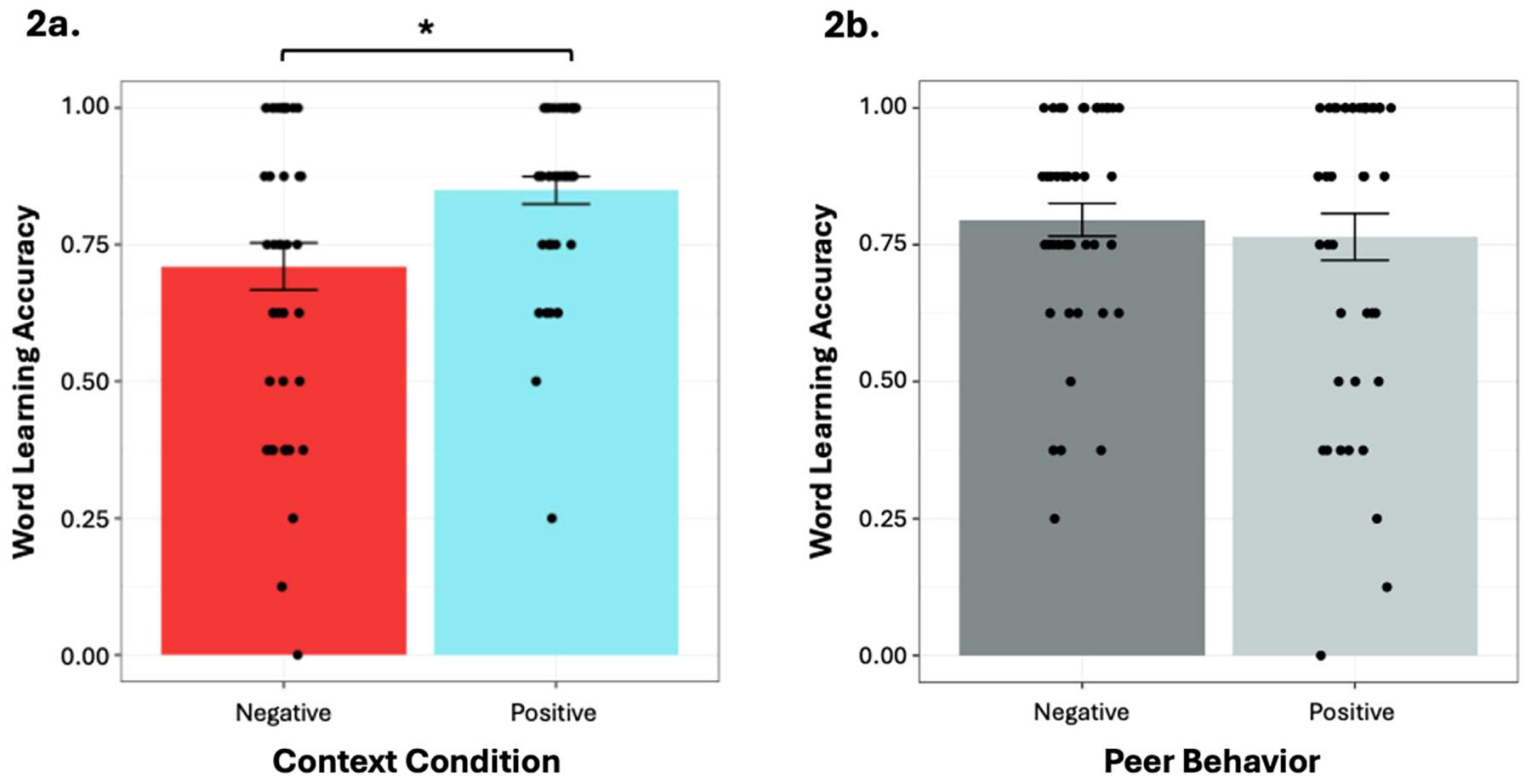
Word learning accuracy by (a) Context and (b) Peer. Children learned significantly better in the Positive Context than the Negative Context, but there were no Peer effects.

The main effect of Peer revealed that there was no difference in accuracy for words taught by the Positive Peer (*M =* 0.76, *SD* = 0.28) compared to the Negative Peer (*M* = 0.80, *SD* = 0.20), *b* = ‐0.031, *SE* = 0.050, *F*(1,42) = 0.39, *p* = 0.54 (see Figure 2b). Therefore, inconsistent with our hypothesis, we did not find any evidence that the social behavior of individual peers influenced word learning accuracy. Participants learned equally well from the Positive Peer and Negative Peer. Additionally, the interaction between Context and Peer was not significant, *b* = 0.097, *SE* = 0.10, *F*(1,42) = 0.93, *p* = 0.34, such that the effect of Peer did not differ by Context or vice versa (see Figure 3).

**FIGURE 3:**
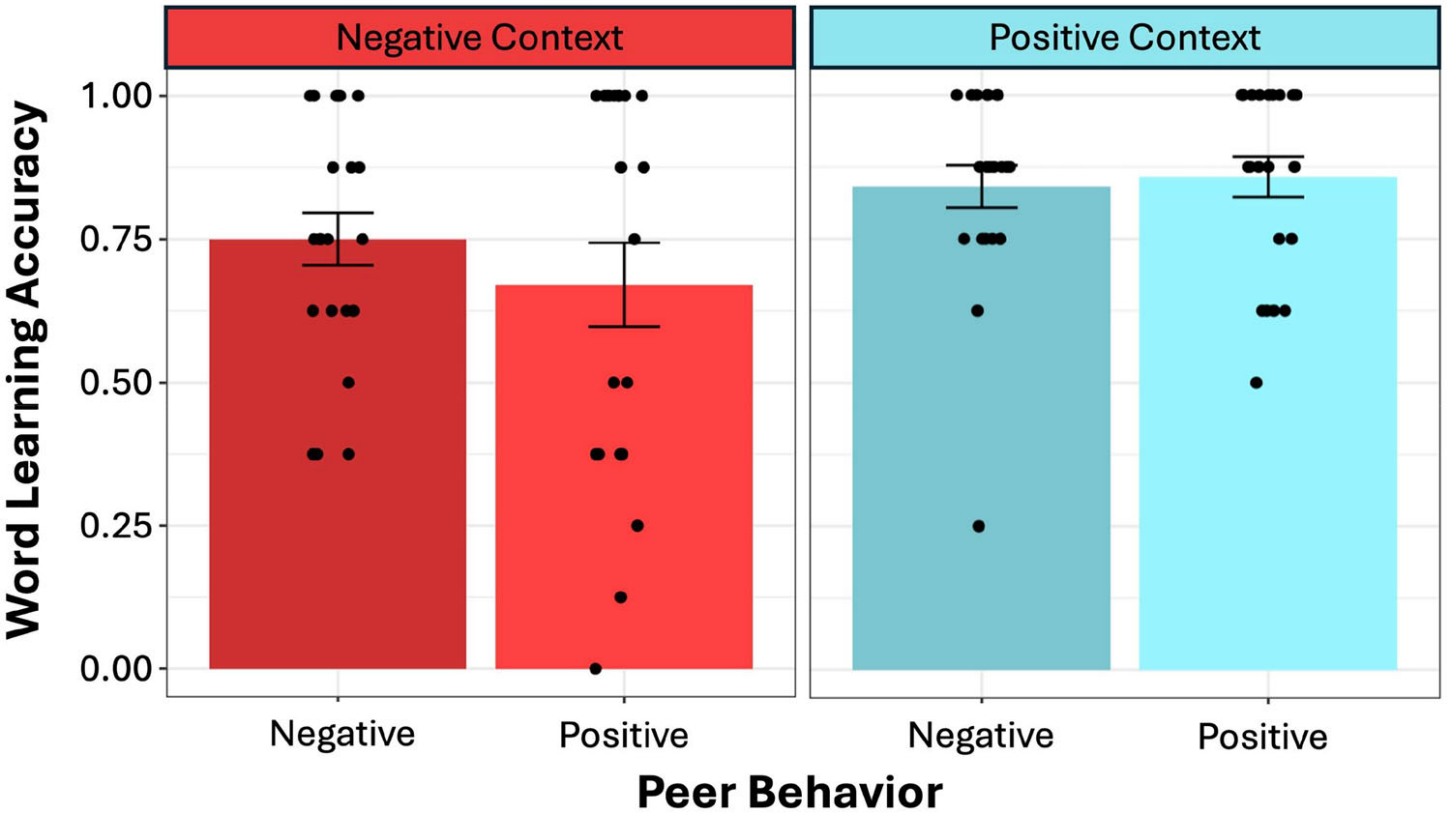
Word learning accuracy by Context and Peer. There was no interaction between Context and Peer.

Lastly, we examined responses to our two additional measures: caregivers’ responses to the demographic survey and participants’ responses to the final question of the task: which friend (between the Positive Peer and Negative Peer) they would want to play with. We collected survey responses from 40 out of our total 44 participants. The majority of participants in our sample had at least one sibling (*N* = 31) and experience in some kind of classroom‐based childcare setting (i.e., center‐based daycare, preschool, or 4k; *N* = 36). Therefore, in general, children in our sample had experience with the classroom context used in our task. However, we did not have sufficient variance in these measures to explore whether differences in these experiences might have influenced children's performance on our task or their sensitivity to our manipulations. Since the sample included a wide age range (36‐60 months), we also examined the effect of participant age by adding age as a predictor in the interaction model, but did not find any significant effects of age on learning. Finally, participants’ responses to the question of which peer they would want to play with did not relate to the effect of our peer manipulation. In a linear‐mixed effects model regressing accuracy on Peer, Peer Preference, and their interaction (including a by‐subject random slope for Peer), the interaction was not significant, *b* = ‐0.048, *F*(1,42) = 0.22, *p* = 0.64, suggesting that participants’ expressed peer preference did not relate to their learning outcomes from the Positive Peer compared to the Negative Peer. Furthermore, only about half (*N* = 23) of the sample choose the Positive Peer in response to this question, suggesting that participants’ expressed preferences may have been driven by factors unrelated to our manipulation.

## 4 Discussion

The aim of the current study is to begin to establish the impact of peers’ social behavior on children's ability to learn words from them. Participants interacted with an animated video of children in a classroom. We manipulated the affective valence of the overall learning context in two between‐subjects conditions— Positive and Negative Context—and we manipulated the social behavior of individual peers within‐subjects—a Positive and Negative Peer. Children were then taught labels for novel objects by both peers. Our results revealed more accurate word learning in the Positive Context condition than the Negative Context condition, suggesting that the positive group‐level context best supported preschoolers’ word learning. However, participants learned equally well from the Positive and Negative Peer. It is important to note that while we found better word learning in the Positive Context condition than in the Negative Context condition, on average, children did learn words above chance in both conditions. Therefore, it is not the case than children *cannot* learn in negative contexts, but rather than they learn *better* in positive contexts than negative contexts.

The results of this study suggest that the emotional valence of the learning context as a whole may play a particularly important role in facilitating the word learning process, perhaps even beyond the effects of the social behavior of the individuals providing language learning input. We know that infants and young children prefer to learn language from adults who display positive behaviors and that these positive interactions can even prime future learning (Singh et al. 2002; Kim and Johnson 2014; Breitfeld et al. 2024). However, it is also important to consider that children, especially in the preschool years, are not just interacting with single individuals. Many 3‐ to 5‐year‐olds are entering group childcare settings and beginning to interact with peer groups. Therefore, the dynamics of behavioral affect do not just operate on the individual‐level, but also at the group‐level, to create either positive or negative social contexts. Our context manipulation may have been particularly salient for children at this stage of development when they are first entering peer group contexts and beginning to develop group‐based social preferences (Kinzler and Spelke 2011). Indeed, previous research on selective word learning suggests that preschoolers often privilege group membership information above reliability information about specific individuals when selecting who to learn from (Elashi and Mills 2014; MacDonald et al. 2013). Though we cannot identify the specific mechanisms influencing word learning in the current paradigm, it is possible that the context manipulation at the beginning of the task may have increased participants’ engagement in the Positive Context condition, but led participants to disengage in the Negative Context condition. This account is consistent with recent proposals that learning‐supportive social behaviors like IDS and contingency support language learning by heightening children's attention (Masek et al. 2021; Nencheva and Lew‐Williams 2022).

It is somewhat surprising that our results did not demonstrate an effect of peers’ individual‐level behavior on word learning. One possible explanation resides in the timing of the manipulations. Because participants were introduced to the context manipulation before the peer manipulation, they may have viewed the behavior of the individual peers as additional data points contributing to the overall trend of a positive or negative group context, rather than attending to the differences in behavior between the Positive and Negative Peers as individuals. In other words, participants may not have perceived the context and peer manipulations as separate. Second, participants may have attended to the difference in behavior between the Positive and Negative Peer, but not based their learning on these differences because the two peers did not provide conflicting labels (Vanderbilt et al. 2014). Previous research has suggested that when speakers provide conflicting labels for an object, children choose to endorse labels provided by speakers with positive affect over labels provided by speakers with negative affect; however, when speakers’ labels are not in conflict, children learn equally well from speakers with positive, neutral, and negative affect (Ma et al. 2022; Ogren and Sandhofer 2021).

Another possibility for why our results support an effect of overall context but not an effect of individual peer behavior could be due to differences in the target, or recipient, of the positive and negative affect between the Context manipulation and the Peer manipulation. In the Context manipulation, the children in the video expressed their positivity or negativity in response to the teacher. However, in the Peer manipulation, the children expressed their positivity or negativity in response to another peer. Perhaps the difference between the positive and negative social behavior was more salient when directed at the teacher, an authority figure, than when directed at a peer (Underwood et al. 1992).

At the end of the study, participants were explicitly asked about which of the children (the Positive or Negative Peer) they would prefer to play with. We found that only about half (*N* = 23) of the sample chose the Positive Peer in response to this question. Importantly, this result provides some evidence to suggest that participants were not attending to the differences in behavior between the Positive and Negative Peer, and therefore our Peer manipulation may not have been salient enough to influence word learning. Alternatively, despite attempting to control for baseline preferences for specific characters by counterbalancing which child was the Positive Peer and which was the Negative Peer across our sample, it could be the case that participants’ responses to this preference question were motivated by factors unrelated to our manipulation. It is also possible that some children found the Negative peer enticing or novel, which could explain both the peer ratings and the lack of peer effects on our word learning results.

Furthermore, while the goal of the current study was to simulate a classroom setting, there may be important differences between children's learning in a tablet study compared to an in‐person interaction. For example, participants in this study only observed interactions between peers rather than engaging in these interactions themselves. Word learning may be differently affected after observing vs. participating in negative and positive interactions with peers. In particular, differences in positive or negative behavior may be more salient when directed towards the child themselves. That is, if the positive and negative peer in the current study had been addressing participants directly, we may have seen an effect on learning. However even in the current paradigm where participants simply observed interactions, we still find an effect of overall social context on word learning. The current results underscore the value of continuing to examine these dynamics in real‐world settings to better understand word learning outcomes.

Finally, while our results examine average trends across children, future research should continue to explore potential individual differences in children's sensitivity to others’ social behavior. We did not have sufficient variance in our individual difference measures of participants’ previous experience with peers (siblings, group childcare) to explore whether these differences might have influenced their performance on our task or their sensitivity to our manipulations. However, it could be the case that some children may learn words equally well from all speakers, regardless of behavior, while other children may have strong preferences for learning from positive speakers, and these differences might be able to be explained by differences in children's prior social experiences.

More broadly, the results of the current study contribute to a growing literature exploring features of children's learning contexts that relate to variation in word learning outcomes. A variety of factors—linguistic, semantic, visual, physical, social, and so forth—can make it easier or harder for children to learn words from a given interaction (e.g., Ackermann et al. 2020; Benitez and Saffran 2018; Breitfeld and Saffran 2024; Fitch et al. 2020; Pomper and Saffran 2019; Reed et al. 2017). Furthermore, both features of learning opportunities themselves, but also of prior experiences, can impact children's ability to learn words (e.g., Breitfeld et al. 2024; Kartushina et al. 2021; Shneidman et al. 2009; van Rooijen et al. 2019). These factors have the potential to vary across children and across time as well as across cultures and communities. The current study suggests that interactions with peers is another important factor of children's experiences that contributes to word learning potential.

Our results extend previous research suggesting that children learn better from positive individuals to suggest that positive group‐level social contexts also boost word learning compared to negative group contexts. As children enter the preschool years, their learning contexts expand from one‐on‐one interactions with adults, to interactions with larger peer groups. Preschoolers successfully learned words on average across all conditions and learned equally well from a negative and positive peer, but importantly, they learned words significantly better in the Positive Context than in the Negative Context. Therefore, exploring specifically how social interactions with peer *groups* impact children's word learning provides a more complete picture of early language learning environments. Our results suggest that fostering positive peer group contexts may be a key factor in boosting preschoolers’ word learning success.

## Author Contributions

**Elise Breitfeld**: writing – original draft, methodology, writing – review and editing, data curation, formal analysis, investigation, visualization, software, supervision. **Courtney N. Snoberger**: conceptualization, writing – original draft, methodology, writing – review and editing, data curation, investigation, validation, software, funding acquisition, project administration. **Jenny R. Saffran**: methodology, writing – review and editing, project administration, supervision, resources, funding acquisition.

## Conflicts of Interest

The authors declare no conflicts of interest.

## Ethics Statement

This research was performed in accordance with guidelines for ethical treatment of human participants, and the protocol was approved by the University of Wisconsin‐Madison Institutional Review Board.

## Supporting information




**Supporting Information**: desc70294‐sup‐0001‐SuppMat.docx

## Data Availability

The data that support the findings of this study are openly available in the Open Science Framework (OSF) at https://osf.io/zswe3/overview
