# Supplementary material for: Positive Peer Groups Support Preschoolers’ Word Learning
Source: Dev Sci. 2026 Oct 7;29(6):e70294. doi: 10.1111/desc.70294 (PMC13646472; doi:10.1111/desc.70294)
Supplement: Supplementary file 1 [file DESC-29-e70294-s001.docx]

**Positive peer groups support preschoolers’ word learning**

**Supporting Information**

**Exposure Phase Dialogue**

**Positive Context Manipulation (between-subjects).**

Teacher: Hi there! I am Mrs. Fredrickson, and today my class will be doing an art project together.

*Click on the teacher to see what happens next*

Child 1: I am so excited!

Child 2: We are going to make something so cool!

Child 3: I love making art! This is such a fun class!

Child 4: You are going to make something so cool. I can’t wait to see!

Child 5: Thanks, I can’t wait to see what you make too!

Teacher: Alright class let’s get started. We will be working in pairs for this project. You will be using things from around the classroom.

*Touch the table to see what the kids make.*

**Negative Context Manipulation (between-subjects).**

Teacher: Hi there! I am Mrs. Fredrickson, and today my class will be doing an art project together.

*Click on the teacher to see what happens next*

Child 1: I don’t want to do art!

Child 2: We are not going to be able to make anything cool!

Child 3: I hate making art! This is such a boring class!

Child 4: I don’t think you will be able to make anything cool!

Child 5: Well, I don’t think you will be able to make anything good either!

Teacher: Alright class let’s get started. We will be working in pairs for this project. You will be using things from around the classroom.

*Touch the table to see what the kids make.*

**Peer Manipulation (within-subjects).**

Positive Peer: Hi, friend! Aren’t you so excited to work on this art project?

Negative Peer: No, I am not excited. I don’t like art.

Positive Peer: Why aren’t you excited? I think we are both going to make something so cool!

Negative Peer: I am not excited because I know I am not good at art. I don’t think you are very good at art either.

Positive Peer: Well, I still hope you make something cool. I am going to start planning how to build my project.

Negative Peer: Ok, whatever.

Positive Peer: Do you like my plan for the project?

Negative Peer: No, that’s a bad plan. It will never work.

Teacher: Hi, girls! Here are some more things for you to use for your art project. Some of these things you might know, but some of them you might not have seen. Can you help me share what these things are?

*Touch the box to see what the things are.*

**Additional Statistical Analyses**

**Preregistered basic models.** In addition to the interaction model reported in the main text, two single-predictor models were preregistered to test the effects of each manipulation (Context and Peer) separately. The results of these models are convergent with the pattern of results from the interaction model reported in the main text. In both models an offset of -0.5 was applied to accuracy (such that a value of 0 was equal to chance performance) and Context and Peer were both centered (-0.5 for Negative and 0.5 for Positive).

In the first single-predictor model, accuracy was regressed on Context in a linear model with no random effects (as Context was manipulated between-subjects and accuracy was calculated at the subject level). The results of this model revealed a significant intercept (*b*=0.28, *F*(1,42)=119.3, *p*<.0001) and a significant effect of Context on accuracy (*b*=0.14, *F*(1,42)=7.38, *p*=.0095). Consistent with the results reported in the main text, this model suggests that participants learned the words above chance on average across the Positive Context and Negative Context and that accuracy was significantly higher in the Positive than the Negative Context.

In the second single-predictor model, accuracy was regressed on Peer in a linear mixed-effects model with a by-subject random slope for Peer (as Peer was manipulated within-subjects). The model with full random effects (a by-subject random intercept and random slope for Peer) did not converge, so the by-subject random intercept was removed. The results of this model again revealed a significant intercept (*b*=0.28, *F*(1,43)=103.92, *p*<.0001), but no significant effect of Peer on accuracy (*b*=-0.03, *F*(1,43)=0.40, *p*=.53). The results provide further converging evidence that learning was above chance on average across the manipulations; however, participants learned equally well from the Positive and Negative Peer.

**Logistical regression approach.** In addition to the preregistered linear mixed-effects model approach reported in the main text in which accuracy was calculated at the subject level, an alternative approach is to use a logistic mixed-effects model and maintain accuracy data at the trial level (0 or 1). The results of a logistic mixed-effect model regressing accuracy (0 or 1 at the trial level) on Context, Peer, and their interaction yields convergent results with the linear mixed-effects model run on the subject-level accuracy data reported in the main text. Namely, we find a significant intercept and a significant effect of Context, but no effect of Peer nor an interaction between Context and Peer (see model results below).

| **Predictor** | **Estimate** | **SE** | **z-value** | ***p*** |
| --- | --- | --- | --- | --- |
| Intercept | 1.84 | 0.31 | 5.92 | <.0001 |
| Context | 1.02 | 0.43 | 2.38 | .0171 |
| Peer | 0.35 | 0.47 | 0.74 | .459 |
| Context:Peer | 0.77 | 0.86 | 0.89 | .369 |

**Model comparison approach.** A final exploratory approach to examine the effect of the two manipulations is model comparisons to determine whether the manipulations explain significant variance when added to the model as predictors. Consistent with the results reported in the main text, the interaction model (predicting accuracy from Context, Peer, and their interaction) explains significantly more variance than the null model (intercept-only), χ²(3) = 8.62, *p*=.037. Furthermore, this is primarily driven by the addition of Context as a predictor. The Peer-only model does not explain more variance than the intercept-only model, χ²(1) = 0.41, *p*=.52; however, the full model does explain more variance than the Peer-only model, χ²(2) = 8.05, *p*=.018.
